# Contributions of a time use perspective in community mental health practice: a scoping review

**DOI:** 10.3389/fpsyt.2024.1461705

**Published:** 2024-10-11

**Authors:** Ellie Fossey, Nastaran Doroud, Carol Ann Harvey, Carolyn Dun, Danielle Hitch, Louise Farnworth, Terry Krupa

**Affiliations:** ^1^ Department of Occupational Therapy, Monash University, Melbourne, VIC, Australia; ^2^ Department of Nursing and Allied Health, Swinburne University of Technology, Melbourne, VIC, Australia; ^3^ Department of Psychiatry, The University of Melbourne, Melbourne, VIC, Australia; ^4^ Northern Area Mental Health Service, Melbourne, VIC, Australia; ^5^ Occupational Science and Therapy, Deakin University, Geelong, VIC, Australia; ^6^ Department of Occupational Therapy, Western Health, Melbourne, VIC, Australia; ^7^ School of Rehabilitation Therapy, Queens University, Kingston, ON, Canada

**Keywords:** time use, participation, recovery, occupational therapy, mental illness

## Abstract

**Introduction:**

Time use is an important indicator of health and well-being. Exploration of time use can provide in-depth information about individuals’ activity patterns including routines and structure, the experience of activities, personal priorities and challenges. People experiencing severe mental illness may be at particular risk for time use patterns associated with poor health and wellbeing.

**Methods:**

This scoping review aimed to identify and map the evidence about how a time use perspective informs assessment and intervention in community mental health practice. Electronic databases and hand-searches were used to identify relevant studies involving people with severe mental illness, and focused on time use applications in practice. Twenty-nine studies were included in this review, data-extracted and synthesized with reference to the review question.

**Results:**

Of the twenty-nine identified studies, seven described development and psychometric testing of time use assessments; twelve used time use tools to measure outcomes; and ten described or evaluated time use intervention approaches. The identified time use assessments typically involved retrospective diaries completed before or during structured interviews, and an Experience Sampling Method using smart technology to gather activity data in real time. Both psychosocial and occupational interventions used time use to measure outcomes relevant to activity engagement, social functioning, and personal recovery. The identified time use interventions originated in occupational therapy; included structured manuals and workbooks to enable reflection on daily time use; individual or group sessions to collaborate in identifying priorities, goal planning and supporting desired changes to activity patterns. These interventions were viewed favorably overall, with improved activity engagement, quality of life, and personal recovery reported.

**Discussion:**

Time use assessments evaluate outcomes of importance to personal recovery and community inclusion. Time use interventions address activity patterns associated with poor health and wellbeing, using collaborative and supported activity engagement approaches, and contribute to emerging evidence on interventions that support personal recovery. Furthering peer involvement in developing and delivering these interventions will bring important insights to time use practice and research, while the social forces that marginalize or constrain activity participation for people with severe mental illness also need to be addressed to advance time use and other approaches that aim to support community inclusion.

## 1 Introduction and background

How people spend their time has long been recognized as an important indicator of health and well-being in various disciplines. Early in the 20^th^ century, Adolf Meyer (1), a psychiatrist, espoused that the active and purposeful use of time had the potential for both restoring and maintaining health; and that the ways in which individuals used and organized their time reflect adjustments to daily living demands. Such principles informed the beginning development of the occupational therapy profession in the 1920s, and the conceptual roots of the recovery movement (2). Time use is of continuing interest in occupational therapy principally as a means to understand and address disrupted and restricted activity patterns that contribute to individuals’ risk for ill-health (3). Further, as part of their public health strategies, many nations routinely collect time use data to better understand and influence the health and well-being of the general population (4). This emphasis on time use is grounded in the belief that various features of activities – such as frequency, meaning, repetition, duration, and variety – within a temporal context can either promote or compromise health. Public health applications of time use specifically target populations at risk for poor health and well-being due to problematic time use patterns with wide-ranging social impacts (4). As illustrative examples, these include adolescents’ time use patterns associated with risk-taking, problem behaviors and mental health (5, 6); differences in time use among mothers and fathers (7); and health and well-being concerns emerging from time use patterns of unemployed youth, retirees and older adults such as a predominance of sedentary activity (8–12).

Ongoing health conditions, associated functional limitations and disability have also been linked to time use patterns that further contribute to poor health and wellbeing (13–16). For instance, a number of time use studies involving community dwelling people with severe mental illness have reported imbalanced activity patterns that show limited time spent in productive activities (such as work, parenting, education) and most time spent in sedentary or passive leisure activities (e.g. watching TV), self-care (e.g. eating and sleeping) and alone (17–20). Health and well-being concerns about such time use patterns include the limited variety of activities that individuals experience, reduced opportunities for community contributions and participation, weakened social connections and overall social marginalization (17).

Time use is an important way of conceptualizing and understanding engagement in activities and their impacts on health and wellbeing for people with severe mental illness. It provides insight into how people structure their time, their priorities, the social and spatial contexts of their lives and key barriers they face (21). Time use contributes to understanding how activity patterns are related to community inclusion and adjustment (17). Time use practice approaches offer a collaborative way to map day-to-day experiences, enabling individuals to reflect on the impacts of their daily activity experiences on their health and wellbeing (13, 22). Furthermore, opportunities to engage in personally and socially meaningful activities have consistently been identified by people living with severe mental illness as an important element of personal recovery, a process that differs individually but broadly involves creating a life of well-being and meaning with or without the continuing presence of illness (23–26). The potential of engagement in self-chosen activities to support personal recovery lies in their capacity to create conditions for building hope, meaning and purpose in life, developing social connections in communities of choice and ways of self-managing one’s health in everyday life (27–29). As such, the extent to which time use focused approaches support people in their recovery should be considered an important outcome (3).

This paper focuses on time use as a practice approach for working with people with severe mental illness to construct self-chosen, meaningful and health-enhancing activity patterns and thereby to support recovery and wellbeing. To the best of our knowledge, the available evidence to guide time use focused practice approaches to these issues in mental health care has not previously been mapped or synthesized. A scoping review approach was considered appropriate for this purpose, given the authors’ understanding that the evidence base in this area is diverse in both research methodologies and disciplinary context (30). The concept of time use has been used in public health and social sciences as an indicator of productivity, mental health, happiness and quality of life; and to understand the impact of disability at individual and population levels (4, 15). Describing the extent, range and content of the available evidence will provide a clearer basis for practices informed by a time use perspective, as well as rigorous guidance for ongoing practice developments and research directions.

### 1.1 Aims and objectives

The overall aim of this scoping study was to synthesize evidence on practice applications of a time use perspective to support the personal recovery of people with severe mental illness. The specific review question was: *‘How does a time use perspective inform assessment and interventions within community mental health practice?’*

## 2 Materials and methods

This scoping study utilized the method originally developed by Arksey and O'Malley (31) and further elaborated by Levac et al. (32). It includes six steps: a) identifying the research question; b) identifying relevant studies; c) study selection; d) charting the data; e) collating and synthesizing the results; and f) consultation. While a protocol was not registered for this scoping review, the reporting of this scoping review conforms to the PRISMA Extension for Scoping Reviews (PRISMA-ScR) (33).

The research team consisted of senior academics and community mental health clinicians with substantial research and practice experience of time use assessment and intervention. The team identified the review question from their professional knowledge of gaps in current understanding and applications of a time use perspective in mental health practice. The review began with a preliminary search on Google Scholar guided by the SPICE framework (34) to assist in identifying the key concepts (**Table 1**).

**Table 1:** SPICE framework terms.

Category	Terms
Setting	Community
Perspective	adults, older adults, patients, consumers, service-users, clients, mental health, mental illness, psychiatry
Phenomenon of Interest	Time-use, engagement in activities
Comparison	Not applicable
Evaluation	Quantitative, qualitative and mixed methods studies reporting primary research

The final search strategy was developed in consultation with a university librarian with expertise in health databases; and tested through a trial search of the EBSCOHOST platform. Examples of the search terms included “Mental disorder*” or “Psychotic disorder*” or “Schizophreni*” or “Mental illness*” or “Psychosocial disability*” or “Psychiatric disorder*” or “Psychiatric patient*” or *“Mentally ill”;* AND *“Time use”* or *“time use”* or *“Use of time”* or *“Occupational balance”* or “Occupational engagement*”. **Supplementary Table S1** in **Supplementary Materials** presents the search strategy.

The search strategy included limiters to ensure sources were peer-reviewed, published in English and included adults (age 18 or older). Book chapters, conference papers, theses, pre-prints, study protocols and letters were excluded. No time limits were set, so as to locate any studies focused on time use applications in community mental health practice. Nine databases were selected to identify studies meeting the inclusion criteria, including five databases within EBSCOHOST platform (Academic Search Complete; CINAHL Complete; Health Source – Nursing/academic; Social Work abstracts; SocINDEX) and four other databases: PsycInfo, PsycArticles, Scopus, and Web of Science. The database searches were last updated in June 2024.

After duplicate removal, 487 studies were imported to Covidence for screening. Two authors (ND, EF) independently screened all studies, with disagreements discussed and resolved by consensus in regular meetings. Title and abstract screening excluded 345 studies, leaving 142 sources to proceed to full-text review. At this stage, the reference lists of included studies were searched for further potentially relevant sources. Google scholar was also used for citation tracking and searches of key authors or journals to retrieve studies that may have been missed in the database searches. These processes identified a further 20 potentially relevant studies for full text screening. Therefore, the full text of 162 studies were reviewed for eligibility, resulting in 29 studies being included in this review based on the following criteria:

a) studies were of time use assessments, time use as an outcome measure or time use focused interventions (i.e. studies describing time use patterns, time spent in specific activities such as employment, internet-use, or physical activity, or investigating time use associations with clinical and socio-demographic factors were excluded);

b) study participants were people with severe or persistent mental illness, defined as a primary diagnosis of schizophrenia, schizoaffective disorder, bipolar disorder, or other severe and enduring psychotic disorder (29) (i.e. studies involving people with other diagnoses such as depression and anxiety were excluded);

c) studies were conducted in community or outpatient setting (e.g. excluding studies in inpatient or secure settings); and

d) papers reported original studies (i.e. excluding reviews, protocols and secondary analyses).

**Figure 1:**
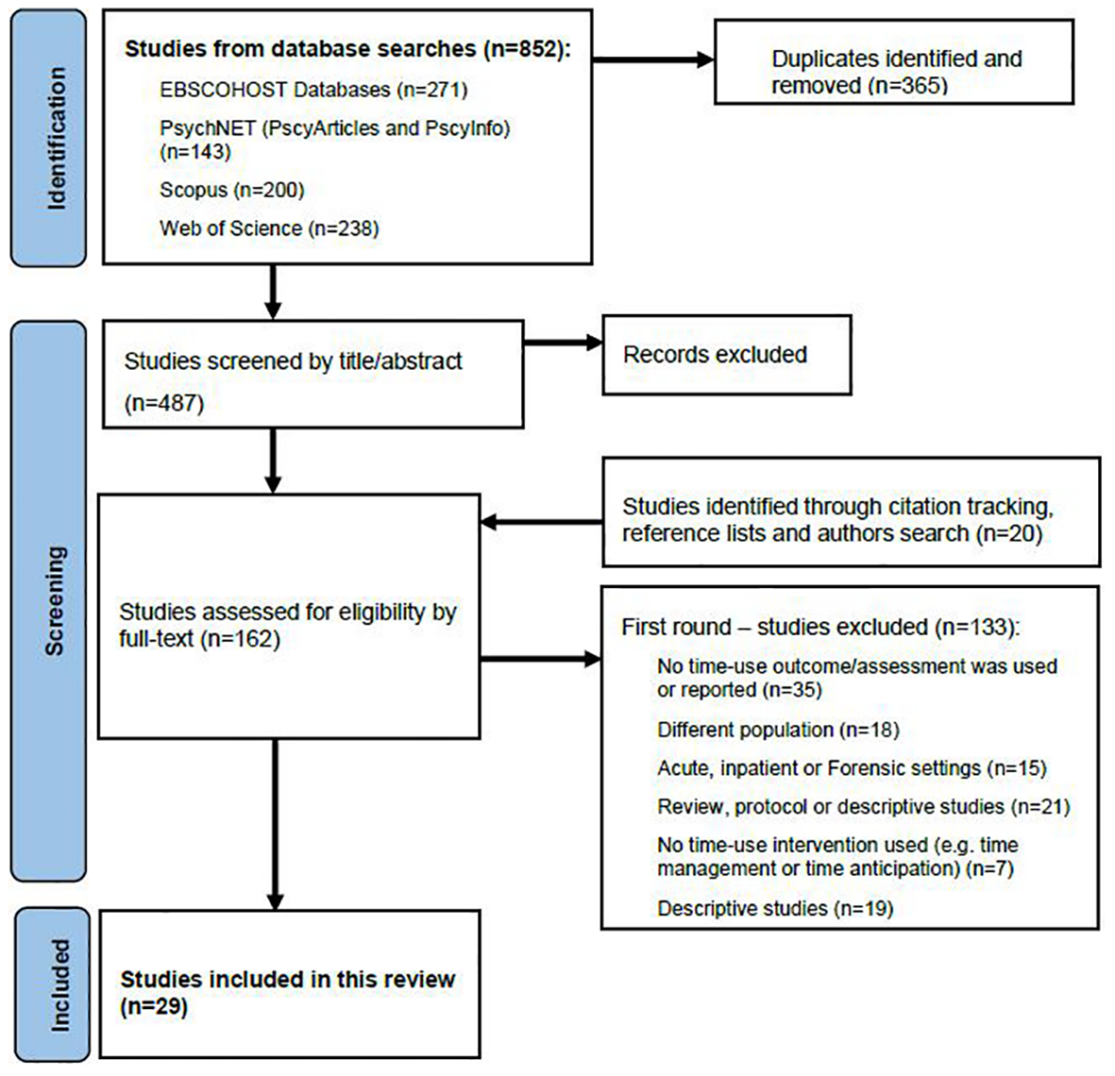
PRISMA flow diagram of the screening and selection of studies.

**Figure 1** illustrates the screening and selection processes, using a PRISMA flow diagram (35). The resulting studies were grouped into three main categories: 1) development and psychometric testing of time use assessments (n=7); 2) studies using time use as an outcome measure (n=12); and 3) studies of time use focused interventions (n=10).

Data were extracted from the 29 studies according to key fields suggested by Arksey and O'Malley (31): authors, location, study aim, methodology and design, and key findings. Additional fields relevant to our review question were used to record details about participants (e.g. diagnosis, gender), time use assessments, outcome measures and interventions, as well as other data collection methods, implications and limitations. The team developed the data extraction template, with all data extracted by a single author (ND) and presented in three separate tables based on the review question: time use assessments, time use as an outcome measure, and time use focused interventions. Within each table, similarities and differences between the studies were compared. For example, similarities between approaches to completing time use assessments, and in the development and delivery of time use interventions, were compiled by two authors (ND, EF), to synthesize the results.

## 3 Results

### 3.1 Study characteristics

The 29 included studies were published between 2006 – 2023. The majority were from Sweden (n=14), followed by the UK (n=7), Canada (n=2), Italy (n=2), Australia (n=2), Denmark (n=1) and Portugal (n=1). Most studies recruited participants from community mental health settings; three studies recruited participants from multiple settings including in-patient, residential and outpatient clinics; and two recruited from supported residential settings. Study methodologies included randomized control trials (n=14), psychometric testing (n=5), qualitative methods (n=5), pilot or feasibility studies (n=3), one correlational study, and one mixed-methods study. Most studies were designed and conducted by occupational therapists (n=20) including four studies that developed or tested time use assessments, and all studies of time use interventions. Others involved the disciplines of psychiatry, psychology and social sciences.

### 3.2 Time use assessments

Seven studies reported on the development, psychometric testing or practice application of time use assessments (**Table 2**). Two of these assessment tools are based on time use diary methods and one uses the Experience Sampling Method (ESM), as described below.

**Table 2:** Studies of time-use assessments (n=7).

Author (year)	Location & Setting	Aim	Design	Participants	Time-use measures (content and structure)	Findings	Implications
Bejerholm et al. (2006) (36)	Sweden; outpatient services	To develop and examine psychometric properties of the POES on measuring engagement in daily activities (part of a larger study investigating daily occupations, health, quality of life and wellbeing)	Cross-sectional correlational; psychometric testing	Time-use diaries of adults with schizophrenia randomly selected from the larger study (n=41); (10 to construct the pilot version of the POES, 4 for content validation, 27 for interrater reliability and internal consistency)	Time-use diary (POES): • Developed from qualitative data and accounts of time-use from people with schizophrenia • Measures time-use during past 24 hours for a weekday • Separated in 1-hour intervals • Four columns: occupation/activity, geographical and social environment, personal reflection • A self-report questionnaire, supplemented by an interview to ensure completion and recall; and to help validity • Completion takes 45 minutes	• POES has relevant content and can be used. Items reflect occupational engagement, severity of symptoms and environment among people with schizophrenia • POES can estimate engagement in real-life activities • Good interrater reliability, high Cronbach’s alpha value, and consistency in testing procedures with exception of one item (initiating performance). • Training and manual for POES users should be developed to ensure consistency	• POES is suitable to use in community-based and client-centered practice. • POES can help assessing life changes, social interactions; and the impact of personal (e.g. impacts of symptoms and medication) and environmental factors (e.g. hospitalization, social isolation). • POES provides an initial step in identifying occupational problems, imbalance between rest and activity, environmental opportunities; and needs. • POES can be used as an outcome measure to evaluate effectiveness of interventions in real-life.
(Bejerholm & Eklund, 2006a) (37)	Sweden; outpatient services	To examine the validity of the construct underlying the POES	Cross sectional correlational; psychometric testing	Time-use diaries of adults with schizophrenia randomly selected from the larger study (n=41); and interpreted by occupational therapists (n=12)	Time-use diary (POES) (further developed from 36): • Includes 9 items: 1) daily rhythm of activity and rest, 2) place, 3) variety and range of activities, 4) social environment, 5) social interactions, 6) interpretations, 7) meaningfulness of activities, 8) routine, 9) initiating performance. • Each of the items is rated and plotted on a graph to demonstrate a profile.	• Basic construct validity was established; moderate relationship between POES, functioning and satisfactions • Items with strong correlation were: ‘Daily Rhythm’, ‘Variety and range’ and Activity Level’ • POES provides information on clients’ real-life functioning	• POES can supplement symptomology to understand wellbeing in real-life context, extent of engagement, impact of clinical symptoms on client’s day-to-day life, imbalance between rest and activities, and meaning • POES can help goal setting.
(Bejerholm & Lundgren-Nilsson, 2015) (38)	Sweden; outpatient units	To establish the internal construct validity of the POES using the Rasch measurement model	Cross-sectional; Psychometric testing study using	Adults with severe mental illness (n=192)	Time-use diary (POES) (see 36)	• POES has internal construct validity and represents occupational engagement, rhythm of activity and rest, routines, meaningful occupations and involvement in social environments. • High reliability and acceptable overall person-fit but some misfit of items (‘interpretations’ and ‘initiating performance’ were the most difficult items; place the least difficult). • Communication and interaction skills may be associated with engagement in social environments	POES can inform evidence-based practices; and help to identify social engagement, and communication skills
Jolley et al. (2006) (39)	UK; Part of a larger study; inpatient and outpatient settings	1. To validate a simplified time budget measure of activity in psychosis; 2. To study the relationship between time-use and symptoms of psychosis, distress and affect	Descriptive cross-sectional design; Psychometric testing	Adults with a current diagnosis of non-affective psychosis; (n=276)	Time budget measure: • Diary for a typical week completed in a structured interview; • 4 time blocks for each day (28 time blocks overall); • Rated from 0-4 based on activity engagement, diversity, complexities and demands, motivation and planning; and social contacts	• The validity of the time-use measure was supported as an indicator of social functioning. • Time-use scores varied widely; most participants were engaging in passive, or active but simple and brief activities (rating 1 or 2). • Moderate relationships were found between time-use measure, social functioning and symptoms • IQ, negative symptoms and distress were identified as a predictor of time-use in regression analysis • No correlations between demographic variable and the time-use.	The time-use budget may be used to identify social functioning and clinical symptoms.
(Larivière et al., 2017) (40)	Quebec, Canada; Community settings (not indicated explicitly)	1. To translate the POES into French; 2. To establish the interrater reliability of the French version	Cross- sectional; Psychometric testing	Adults with schizophrenia or schizoaffective (n=23)	The French POES (see above, 36): • French POES interpretations: i) 9-18=low engagement; 19-27=moderate engagement; 28-36=high engagement; • Analysis of occupational balance to demonstrate under-/over- occupied or good balance.	• French POES mean scores ranged 19-36 (moderate occupational engagement on average). • High interrater reliability, with lower agreement on social environment, interpretation and meaningfulness of activities. • Some terms and expressions needed further verification or clarity in the French POES	• French POES can be used as a structure for clinicians to measure time-use, and a supplementary tool to enable the person to reflect on their activity engagement. • POES can be used in psychosocial rehabilitation to gain understanding about the individual and to tailor interventions based on their needs.
(Cristina Zarbo et al., 2022) (41)	Northern Italy; Psychiatric Residential Facilities and outpatient settings	To investigate adherence to and usability of 7-day monitoring with ESM and Actigraphy among a sample of individuals with SSD and paired healthy controls	Mixed-method convergent parallel study	Patients with SSD in Psychiatric residential facilities (n=22) and outpatient settings (n=20); and healthy matched individuals (n=26)	Smart-phone ESM: • Brief questionnaire about current activities and mood on a smartphone 8 times a day for 7 consecutive days • Actigraphy to monitor physical activity and sleep (e.g. steps, activity intensity, sedentary time and sleep)	• Lower usability and response rate of mobile ESM in residential participants • Significant negative correlation between usability and adherence to actigraphy • Qualitative findings on usability of ESM and actigraphy: 1) Positive and negative effects of monitoring 2) Factors influencing adherence: motivation, technical features and differences of the both devices, notifications and interruption with daily activities, duration of monitoring, residential rules or setting, number of days and hours of monitoring. 3) Emotions and mental states (e.g. negative and positive emotions such as annoyance, discomfort, interest, pleasure and satisfaction) 4) Advise (on device features, advise related to time)	• The e-monitoring approaches (ESM and actigraphy) have a potential for clinical practice particularly for residential settings (e.g. for remote monitoring during COVID-19 pandemic) • Optimizing the technical features of ESM and actigraphy based on participants’ concerns is important • Training on the use of devices, and positive aspects of devices can be reinforced by staff to assist with self-monitoring, self-reflection, confidence and feeling supported. • Not suitable for people at risk of developing positive symptoms (e.g. delusion of control).
(Zarbo, Zamparini, Nielssen, et al., 2023) (42)	Northern Italy; Psychiatric Residential Facilities and outpatient settings (part of a larger study)	1. To compare adherence to EMS among people with SSD in (residents and outpatient) and control individuals; 2. To investigate the patterns, socio-demographic and clinical predictors of adherence	Cross sectional descriptive and correlational	People with SSD (n=131) in residential facilities (n=74) and outpatient settings (n=57); and healthy individuals (n=115)	Smartphone EMS to assess daily time-use and mood: • brief questionnaire about current activity, 8 times a day for 7 consecutive days • notification to questionnaire between 8am-24am; semi-randomized, with a reminder if not responded.	• Overall adherence was 50% for residents and 58% for outpatients. • Lower adherence to ESM among people with SSD compared to control group. • Decrease in adherence in late evening and after 6 days; • Higher self-esteem and collaboration skills predicted higher adherence; • Socio-demographic characteristics, cognitive functioning and other clinical features had no effect on adherence; • Positive symptoms had a negative impact on adherence (e.g. suspiciousness, hallucination and unusual thought content)	ESM method as a potential approach to measure activities, yet low adherence needs to be considered: 1) ESM within waking hours (e.g. 8am to 8pm) and fewer days; 2) Training for participants and staff about the ESM and smartphone approach 3) Improving participants self-confidence on use of technology

POES, Profiles of Occupational Engagement in people with Schizophrenia; ESM, Experience Sampling Method; SSD, Schizophrenia Spectrum Disorders; RCT, Randomized Controlled trial.

#### 3.2.1 Development of time use assessments

Four studies reported the development and psychometric testing of the Profiles of Occupational Engagement in people with schizophrenia (POES) (36–38). The POES was developed by occupational therapists drawing on accounts of time use and occupational engagement from people diagnosed with schizophrenia (43, 44), previous literature and the authors’ clinical expertise.

The POES utilizes a 24-hour time use diary designed for a person to report their activities in one-hour blocks of time for the preceding weekday. It asks the person to record their activities (i.e. what they were doing, and how long for), the place and the social context (i.e. who else was present or if on their own), and a personal reflection or comment about how the activity was experienced. This is supplemented by an interview (typically about 45 minutes) to support the person’s completion of the diary and reflection on personal experiences related to the activities recorded in it. The POES also allows the rating of activity engagement across nine key areas: 1) daily balance of activity and rest; 2) geographical location; 3) variety and range of activities; 4) social environment; 5) social interactions, 6) individuals’ experiences and interpretations; 7) meaningfulness of activities and/or contribution to wellbeing; 8) daily routines; and 9) initiating performance. Each is rated on a 4-point scale (36–38), highlighting personal and contextual factors that contribute to patterns of time use and activity engagement.

The POES was reported to have good interrater reliability, construct validity, and utility in understanding individuals’ level of engagement, wellbeing, functioning and social participation (36–38). A French version developed in Canada also demonstrated sound psychometric properties and utility as a measure of activity engagement in the mental health context (40). Bejerholm and colleagues further suggest the POES is relevant for mental health practitioners to use since it accurately reflects activity engagement in real-life settings and may aid understanding of how not only psychiatric symptoms and treatments (e.g. medication) but also available resources impact the everyday lives of individuals experiencing severe mental illnesses.

Another time diary-based activity measure intended for routine clinical use with individuals living with psychosis was developed by Jolley et al. (39). Their simplified time budget is completed in a structured interview designed to record a person’s activities over seven days in four daily blocks of time, and to support recall of activities and social contacts. Activities are then rated by the interviewer on a 0-4 scale according to their demands (e.g. whether passive, active but simple, or more complex or socially demanding activities). Jolley et al. (39) reported that this simplified time budget is able to discriminate levels of activity, showed stronger associations with symptom distress than symptom severity, and suggested its use as an indicator of social functioning.

The third identified assessment was a smartphone-based Experience Sampling Method (ESM) for assessment and monitoring of daily life experiences, such as activities, mood, sleep, and symptomatology. In a series of mixed-method studies, Cristina Zarbo et al. (20), Zarbo et al. (41) and Zarbo et al. (42) investigated the feasibility of using smartphone-based brief questionnaire to prompt sampling of experiences of current activities and mood in real time at eight intervals daily (between 8am to midnight) over a week. Their findings suggested the ESM as a useful tool for supporting people with severe mental illness to reflect on their activity engagement, and for staff to gain insights into their daily lives in community settings where regular assessment may be challenging. Some participants disliked the technical features of this approach and its interruptions to their daily activities, while the authors also acknowledged that ESM may be experienced as intrusive or distressing in the context of positive symptoms and recommended reduced sampling times, better training and technical support.

#### 3.2.2 Application in practice

The time use assessment methods identified in this review provide varied ways to gather information on time use patterns and influencing factors in real-life contexts including, for example, resources, supports, symptoms, medication and satisfaction (20, 36, 37, 41). They also provide tangible information readily transferable to collaborative priority setting and planning (36, 37). The POES, for example, includes contextual information about places and people with whom activities are completed, from which resources to support change may be identified (36, 37). In comparison, the simplified time budget (39) may be easier to complete than the POES and less intrusive than the Experience Sampling-based approach. Since it focuses more on the level of activity involvement than on dimensions of activity engagement and experience, it may also be less useful to guide interventions. While originating in occupational therapy, the POES could be used by mental health professionals widely to guide person-centered and evidence-based community practice (40). The potential of the POES and ESM methods to support self-reflection also suggests their potential relevance for facilitating the process of personal recovery.

### 3.3 Time use as an outcome measure to evaluate effectiveness of interventions

Twelve studies (including three sub-studies from a larger project) measured time use as an outcome when evaluating the effectiveness of psychosocial and occupational interventions (**Table 3**). These interventions included staff training about activity engagement, its importance for addressing mental illness and approaches to encourage engagement (49); Individual Placement and Support (IPS) (45, 48); cognitive remediation (50); Social Recovery Therapy (SRT) (based on cognitive behavioral strategies) (52–54); a befriending program (56); lifestyle modification (51) and occupational therapy group programs (55).

**Table 3:** Studies using time-use as an outcome measure to evaluate interventions (n+12).

Author (year)	Location & Setting	Aim	Design	Participants	Application of time-use measures	Interventions/approaches	Main findings	Implications
Areberg et al. (2013) (45)	Sweden; Community and vocational rehabilitation settings	To examine effectiveness of IPS on occupational engagement, work motivation, empowerment and QOL among people with severe mental illness	RCT	Adults with long-term psychosis or psychiatric disability; randomly allocated to IPS (n=60) and TVR (n=60) groups	POES completed at baseline, 6 and 18 months via interview	IPS: • Employment specialists (occupational therapists, nurses and a social worker) using the principles of IPS • IPS training and supervision (every fortnight) was provided. • Fidelity was measured: good at 6 months, and excellent at 12 and 18 month. TVR group received vocational services in health and social services including pre-vocational training or work-related activities in sheltered settings.	• No differences between groups in occupational engagement and empowerment at 6 months; • At 18 months, IPS had a positive effect on QOL, work motivation, empowerment and occupational engagement as measured by time-use patterns (engagement in daily activities and community life)	IPS is a valuable process towards personal recovery that encourages connectedness and life satisfaction. Client-centered approaches to employment such as IPS enhances work motivation and empowerment.
Argentzell et al. (2020) (46)	Sweden; Community mental health settings	1) To investigate whether aspects of activity, socio-demographic and clinical factors mediate or moderate changes in recovery; 2) To explore the possible effect of the BEL intervention compared to standard OT intervention on personal recovery	Part of an RCT (47)	Adults with mental illness assigned to BEL group (n=133) and SOT (n=93)	• POES self-report version; • SDO-OB: four segments including work and study, leisure, home tasks, and care of self; with yes/no options to measure satisfaction and activity balance.	BEL intervention (see 47): • A manualized group- and activity-based program with 14 sessions over 16 weeks (including 2 booster sessions) • Led by trained occupational therapists • Topics were on everyday life, meaningful activities, motivators, healthy living, work-related and social activities • using an educational approach and homework • encourages reflecting on the current life, goals and strategies to reach them; and engagement in real-life activities SOT: • Group and individualized sessions including activities (e.g. arts, crafts or creative activities, daily living and social skills).	• The mediators included: activity engagement, activity level and balance, satisfaction with activities, psychosocial functioning, symptom severity and sense of mastery. • Strongest mediators were activity engagement and mastery, followed by satisfaction and symptoms. • No difference between the BEL and standard OT groups in recovery • Participants with diagnosis of depression/anxiety reported lower recovery scores.	To support recovery, interventions should facilitate meaningful activity engagement to enable control and independence
(Bejerholm et al., 2015) (48)	Sweden, Community mental Health settings	To determine effectiveness of IPS on vocational outcomes and community integration	RCT	People with psychosis or a psychiatric disability (n=120)	Time-use assessed by the POES as primary outcome: • Time-use for a yesterday with 1-hour intervals • Assessed time-use in 9 items on a 4-point scale (see 36) • Data were collected at baseline, at IPS completion (18months), and 6 and 18 months follow-up.	IPS: • IPS training provided to 3 employment specialists, plus supervision every 2 weeks throughout the trial • The employment specialists, a steering committee, a process leader and a supervisor formed the IPS organization with regular meetings during the study • IPS was integrated with the mental healthcare service with good to excellent fidelity. • Control group received TVR including prevocational training in sheltered settings; and clubhouse activities	• Attrition rate was 28% with no difference between the participants who left and stayed at 6 months follow up; • At 6-months no significant differences between the groups in vocational outcomes. • At 18-months, IPS was more effective in gaining competitive employment, working hours and weeks, job tenure, income and rapidity of gaining employment. • 90% in IPS group worked or had an internship compared to 24% in the control group who stayed mainly in prevocational and segregated settings. • TVR group had increased psychosis and depressive symptoms.	The IPS is effective in gaining employment within a shorter period of time, more days and weeks at employment and higher income.
Cardoso et al. (2017) (49)	Portugal; residential mental health units	To assess the efficacy of a staff training to improve service users’ engagement in activities	Cluster RCT	Adults with schizophrenia randomly allocated to control (n=84) and intervention group (n=73)	TUD as a primary outcome: • Completed retrospectively in a structured interview; • Recorded in morning, noon, afternoon, and evening time periods; • Rated on a scale of 0-4 for each item with a maximum possible score of 112 • Higher scores reflect higher and more complex engagement in activities. • Completed at baseline, 4 and 8 months.	Staff training provided knowledge and strategies on impact of mental illness (e.g. cognition and motivation), importance of activity engagement, ways to engage service-users in activities, barriers and action planning. Training was provided to nurses and unqualified unit staff, delivered by an Occupational therapist, an activity worker and a user expert.	• Staff training helped in improving service users’ engagement, but effectiveness was not significant. • The intervention may be more effective for younger service users who had higher level of engagement at baseline • Service users with long-term stay may be resistant to change • No significant improvement in QOL, or service-users’ experience of the service.	The training may be useful to increase staff knowledge about aspects of care, but did not lead to long-term results.
Cella et al. (2019) (50)	UK; community clinics	To explore correlations between cognitive remediation (CR) and therapeutic outcomes	correlational cross-sectional study (part of a larger RCT)	Adults with schizophrenia or schizoaffective, with cognitive difficulties randomized to CR (n=38) and TAU (n=8) groups	TUS: • Time spent in structured activities to assess functioning • Semi-structured interview with retrospective report of the time spent in activities such as work, education, volunteering, leisure, sports, socializing, hobbies, resting, household chores, childcare and sleep.	CR: • Delivered by psychologists using a web-based computerized software • Targeted cognition, meta-cognition, strategy use, and transfer of learning. • The tasks included attention, memory and executive functioning. • CR was offered 3/week over 12 weeks, maximum of 40 sessions, 1 hour each	• The number and intensity of tasks were related to the use of strategies • No correlation between errorless learning and the measures of time-use • Therapeutic alliance was correlated to numbers and usefulness of strategies, cognitive and functional improvement	Therapeutic alliance enhances improvements in cognitive and functional outcomes. CR therapists can help transfer of cognitive skills to everyday lives.
Eklund et al. (2020) (51)	Sweden; supported housing	To explore changes in occupational engagement, personal recovery, psychosocial functioning and symptom severity following the AiMH program	Pilot feasibility study	Adult residents with mental illness who received AiMH program (n=29)	POES to assess occupational engagement; completed via interview. Measured at baseline, 6 and 9-months follow up	AiMH: • Informed by two previous interventions: a) a staff program to enrich the facility with meaningful activities; and b) an activity-based lifestyle intervention to support occupational engagement and balance. • Led by an occupational therapist who completed training; • The AiMH included 8 (5 individual and 3 group) sessions, 35-40 minutes each, over 12 weeks; • Themes of the sessions include motivation, senses and coping with stress, activities and health, dreams and goals; • Sessions included psychoeducation, and activity experimentation; • Each participants received €20 to spend in meaningful activities in between of the sessions supported by staff.	• Participation in AIMH improved occupational engagement and personal recovery from baseline to completion of AiMH. • Improvement in occupational engagement remained statistically significant at follow-up (e.g. routine, variations in activities and in social and geographical locations) • Participants’ view on provision of meaningful activities remained unchanged. • Improvements in psychosocial functioning and symptoms severity were not statistically significant. • Participants satisfaction with AiMH was 75% • No correlations between satisfaction with housing and activity engagement	The AiMH can encourage occupational engagement.
Fowler et al. (2009) (52)	UK; secondary mental health services	To evaluate a cognitive behavioral intervention to improve social recovery (SRCBT) for young people with early psychosis	RCT	Young adults with psychosis and social disability allocated to SRCBT (n=35) and TAU (n=42) groups.	TUS as primary outcome to assess functioning: • Completed through face-to-face semi-structured interview; • Measures number of hours spent in activities per week over the past month. • Activities include: work, education, volunteering, leisure, sport, hobbies, socializing, house chores, resting, childcare and sleep. • Hours in constructive economic and structured activities per week are calculated. • Constructive economic activities include: work, education, volunteering, house chores, and childcare; structured activities also include hobbies, sports and leisure. • Completed at 9-month post intervention.	SRCBT: • Combined CBT and vocational case management • 3 stages: 1) assessment and formulation; 2) working towards goals, identifying pathways to meaningful activities, enabling engagement; 3) promotion of activities related to goals and managing symptoms. • Strategies from CBT (e.g. activity scheduling, behavioral experiments and problem solving) • SRCBT included 12 sessions over 9 months delivered by case managers TAU group received active case management by a multidisciplinary team.	• No significant differences in time-use, functioning, psychotic and emotional symptoms, QOL, and needs • Larger trials are recommended.	• The role of case managers in instilling hope and encouraging engagement in meaningful activities is important. • TUS is a sensitive measure to reflect changes in activities
Fowler et al. (2018) (53)	UK; Early Intervention Service	To assess the efficacy of SRT in improving time spent in structured activities.	RCT	Young adults (aged 16-35) with non-affective first episode psychosis and low level of engagement in structured activities (≤30 h/week in TUS) allocated to SRT (n=76) and TAU (n=79) groups	TUS (see 52) to measure time spent in structured activities in the past month to assess social recovery. Completed at 9- months (post intervention) and 15-months (6-month follow-up).	SRT provided in addition to early interventions: • Drawn from SRCBT; • Manualized intervention including 3 stages; • Delivered in minimum of 6 sessions (see 52) ii) TAU received early interventions.	• SRT led to improved engagement in structured activities by 8.1 hours at 9 months (post interventions) and follow-up.	SRT may be useful in improving functional outcomes particularly for individuals less motivated to engage in existing psychosocial interventions.
Fowler et al. (2021) (54)	UK; Community mental health settings	To determine clinical and cost-effectiveness of SRT in young people with non-psychotic severe mental health problems at risk of social disability.	RCT	Young adults (aged 16-25) with non-psychotic severe mental illness allocated to SRT (n=135) and control (n=135) groups	TUS as primary outcome as indicator of social recovery (see 52); Measured 15 months post randomization	SRT delivered in addition to enhanced standard care: • Manualized individual intervention, further developed from SRCBT (52) • Delivered over 9 months in participants’ homes individually and face-to-face; • Include 3 stages: 1) assessment and formulation; 2) working towards goals, identifying pathways to meaningful activities, enabling engagement in vocational or educational activities (e.g. liaison or referral); 3) promotion of activities related to meaningful goals and managing symptoms using cognitive behavioral techniques. iii) Control group received enhanced standard care (i.e. evidence-based treatments)	• General significant improvements in both groups in engagement in structured and economic activities; • No differences in SRT compared to enhanced standard care in engagement in structured activities; • No significant differences in social anxiety, depression, and symptoms severity; • SRT was not estimated cost effective.	Activity engagement (TUS) is a relevant outcome measure. Person-centered interventions for young people are important.
Inman et al. (2021) (55)	UK; community mental health and psychosis teams	To evaluate feasibility of a pragmatic clinical trial (POINTER) for people with psychosis	Exploratory pre-/post-test feasibility	People with diagnosis of psychosis with functional needs (n=20)	TUS as a primary outcome measure: time spent in constructive economic activity and structured activity per week.	Occupational Interventions: • To improve participation in meaningful activities identified by participants; • Program objectives: 1) to assess occupational performance; 2) to formulate occupational needs; 3) to set goals; 4&5) to plan and implement interventions; 6) re-assess occupational performance; 7) review occupational need and goals; 8) discharge from occupational therapy • Provided by occupational therapists in face-to-face and individualized sessions; in participants’ homes or community • Provided 1-2 times per weeks for up to 6 months • The intervention was tailored to each participants based on their needs and goals • Fidelity and adherence were measured, and any modification was reported by occupational therapists	• 14 participants completed the program, and a total of 188 sessions were delivered. • Modifications to the program were made as required (e.g. related to care coordination). • Improvement in HRQOL (health burdens, and general improvement in self-evaluated transition in health), and better health in general. • Majority (71%) of participants were satisfied with the program, and their participation meaningful activities. • Positive outcomes reported in time-use, experience and satisfaction of occupational performance and participation. • TUS and other outcome measures created minimal burden for participants. • ‘Doing Occupational Therapy Research in Practice’ emerged from the data including: 1) recruitment; 2) balancing research and practice; 3) utility of the occupational intervention log; 4) occupational therapy log revealed the intricacies of practice; 5) rating adherence; and 6) outcome measurement procedures.	It is important to use a pragmatic approach to measure feasibility, fidelity and adherence to occupational therapy interventions. POINTER is a valid approach to report individualized occupational therapy interventions.
Priebe et al. (2020) (56)	UK; Community mental health services	To assess effectiveness of a befriending program to reduce social isolation and improve health and social outcomes for people with schizophrenia	Parallel RCT	Adults with schizophrenia and social isolation (<60 min/day in social activities as measured by TUS) allocated to intervention (n=63) and control groups (n=61); volunteers recruited from local community (n=51)	TUS as primary outcome: • Adapted for people with schizophrenia • Measured minutes/day spent in activities. • Assessed at baseline, 1 week and 12 months in the program; and 6-month follow up. (Details on how TUS was completed were not provided in the study).	Volunteer befriending program: • Participants were matched with volunteers based on their interests and preferences; • An activity booklet (list of free or inexpensive activities in the local community) was provided to participants and volunteers; • Volunteer and participants met weekly for 1 year to engage in activities of their mutual interests and preferences • Monthly social events • Volunteers reported occurrence, length and content to the volunteer coordinator. • Control group met with a masked researcher who provided the activity booklet and spoke about the activities to engage in.	• 22% of the participants were not marched to volunteers (5 ineligible, 1 no longer interested once a volunteer become available) • Half of the sample had the expected number of meetings (at least 13) with average duration of 90 minutes. • 15 social events were organized with 6 participants on average. • No significant difference between the intervention and control group in TUS: improvement in both groups in time spent in activities at completion of the program and 6 months follow-up. • Improvement in social contacts after the program and 6-month follow-up in intervention group • No association between the improvements and symptoms, QOL or self-esteem • No association between compliance and primary outcome	Need for flexibility in befriending programs to accommodate needs and changes in preferences over time.
Bjørkedal et al. (2023) (57)	Denmark; community mental health centers	To investigate the effectiveness of an occupational therapy intervention focusing on activity engagement, functioning and recovery co-led by peer worker	RCT	Adults with a psychiatric disability and functional impairments randomly allocated to intervention (n=67) and control groups (n=69)	POES as the primary outcome measure of activity engagement; Competed at baseline and after the intervention	MA&R: • 22 (alternate group and individuals) sessions • Topics included activities, health, recovery and strategies for activity engagement; • Group sessions were facilitated by a peer worker and occupational therapist; • Sessions included presentations, reflective questions, workbook and peer exchange; some sessions included other methods such as stories and photovoice	• Improvements in the MA&R group did not differ significantly with the control group in activity engagement, personal recovery, functioning and QOL	Individualized and peer support are important to consider in interventions.

TUD, Time-use Diary; CR, Cognitive Remediation; TAU, Treatment as usual; POINTER, Participation through Occupational Intervention Effectiveness; Research; TUS, Time-use Survey; HRQOL, Health-related quality of life; IPS, Individual Placement and Support; TVR, traditional vocational rehabilitation; QOL, Quality of life; BEL, Balancing Everyday Life; SOT, Standard Occupational Therapy; SDO-OB, Satisfaction with Daily Occupations and Occupational balance; AiMH, Active in My Home; IPA, Interpretive Phenomenological Analysis; SRT, Social recovery therapy; SRCBT, Social Recovery Cognitive Behavioral Therapy; CBT, Cognitive Behavioral Therapy; MA&R, Meaningful Activities and Recovery.

Six of the intervention studies measured time use as an outcome using a Time Use Survey (TUS). Adapted from the UK 2000 Time Use Survey for general population (58), the TUS is completed via semi-structured interview and asks about time spent over the past month in activities that include work, education, volunteering, leisure, sport, hobbies, socializing, household chores, resting, childcare and sleep. To date, the TUS has been variously used to measure time spent in structured activities (excluding resting and sleep) as an indicator of overall functioning (50, 52, 53); economic and structured activity participation (55); social participation (e.g. places visited and people with whom activities were completed) (56); and time use as an indicator of social recovery (54). In two studies, cut-off scores for social disability (≤30 hours per week in structured activities) (53) and isolation (< 1 hour per day in recreational or social activities) (56) were applied to determine participants’ eligibility for the intervention, although neither reported how the cut-off scores were determined. Fowler et al. (53, 54) found that the SRT was effective in improving engagement in structured and economic activities. Fowler et al. (54) and Inman et al. (55) concluded respectively that the TUS is a relevant and sensitive measure of real-world functioning, and a suitable outcome measure for evaluating individualized occupational therapy interventions.

Based on a similar approach to the TUS, the previously described time budget (39) was used by Cardoso et al. (49) to measure the effectiveness of a staff training program to improve the activity engagement of people with schizophrenia living in residential facilities. They reported improvements in activity engagement, although these were not statistically significant when compared with the control group. Given the time budget measures activity engagement in 4 blocks daily, it may possibly be less sensitive to activity changes than the POES that records activities at 1-hourly intervals.

Five identified intervention studies reported use of the POES (36, 37) as an outcome measure. POES has been used to measure activity engagement and community inclusion outcomes of IPS programs for people experiencing severe mental illness (45, 48), with significant improvements reported with more engagement in a range of activities and community contexts. Further, when used to measure the outcomes of a lifestyle intervention, Active in My Home (AiMH), for people living in supported housing, significant improvements in activity engagement measured by POES and personal recovery measured by QPR (59, 60) between baseline and completion of the AiMH intervention were reported (51). Further highlighting links between activity engagement and personal recovery outcomes, Argentzell et al. (46) used the POES in conjunction with the Satisfaction with Daily Occupations and Occupational Balance (SDO-OB) (61) to identify aspects of activity engagement (e.g. balance and meaning) associated with effectiveness of the manualized Balancing Everyday Life (BEL) intervention (described in the next section). They reported activity engagement, mastery, satisfaction and symptom severity as the strongest mediators of changes in personal recovery following the intervention. Lastly, Bjorkedal et al. (57) used the POES as a primacy outcome in an evaluation of the Meaningful Activities and Recovery (MA&R) intervention, which combines group and individual sessions and is co-delivered by an occupational therapist and a peer worker to encourage activity engagement and recovery through education, reflective questions, peer exchange of ideas, workbooks and creative methods. No significant improvements in activity engagement were found in the MA&R group compared to those receiving standard occupational therapy services, possibly due to similarities between MA&R and the standard service. Overall however, these five intervention studies suggest POES as a useful tool for evaluating the outcomes of interventions designed to enable changes in the nature and quality of time use patterns.

### 3.4 Time use focused interventions

Ten studies reported the development and evaluation of time use interventions, including Action over Inertia (AOI) (62, 63), Balancing Everyday Life (BEL) (47) and the Pathway to Participation (P2P) program (64). All three interventions were developed by occupational therapists and focus on participants’ time use and developing strategies and supports to engage in desired activities (see **Table 4**).

**Table 4:** Studies of time-use focused interventions (n+10).

Author (year)	Location & Setting	Aim	Design	Participants	Outcome measures	Intervention	Main findings	Implications and limitations
Edgelow et al. (2011) (65)	Canada; ACT teams	To pilot evaluate efficacy of an occupational time-use intervention (AOI)	Pilot prospective RCT	People with SMI living in the community recruited from five ACTs (n=24)	• POES to measure activity engagement • Clinical utility was measured using questionnaires developed for this project	AOI: • Designed for community-dwelling SMI to reconnect them with meaningful activities; • Based on occupational balance and time-use, and recovery framework; • Includes elements on self-determination, psychoeducation and cognitive behavioral strategies; • Includes workbook tailored to individuals in 5 sections: 1) determining the need for change; 2) reflecting on the current balance of activities and making rapid changes to engage in activities; 3) education about SMI and occupational engagement; 4) long-term goal planning and support; 5) ongoing monitoring and refining the plan. • AOI was delivered throughout 12 weeks in individual sessions	• No significant differences between treatment and control group in occupational engagement measured by POES (except for sleep time). • The intervention helped participants to reflect on their time-use and activity patterns. • Therapists benefited from the structure and flexibility of the program, but suggested more time in goal setting	• The AOI can be used alongside with other evidence-based recovery-oriented programs to encourage engagement in meaningful activities through reflection and education. • The program duration may have not been enough to see changes in activity pattern. • Follow-up measurements would be helpful in future studies. • Challenges to recruiting participants and attrition rate should be considered in future studies.
Eklund et al. (14) (47)	Sweden; outpatient and community-based settings	To evaluate effectiveness of the 16-week Balancing Everyday Life (BEL) for people with mental illness	RCT	Adults with mental illness who self-report imbalance in daily activities, randomly assigned to BEL group (n=133) and CAU (n=93)	• sociodemographic and clinical data (self-report). • POES (self-report) to measure engagement in productive activities. • SDO-OB to evaluate satisfaction and activity balance. • OVal-pd to measure perceived values of everyday activities. • MANSA to measure QOL. • Rosenberg self-esteem scale. • First item of the MOS SF-36 to measure perceived health. • GAF to measure psychosocial functioning. iv) Completed at baseline, after the BEL (16 weeks) and 6-month follow-up	The BEL: • Developed by occupational therapists. • Group-based program with 5-8 participants. • 12 sessions weekly plus 2 boosters within two-week intervals • The main themes included activity balance, meaning and motivation, healthy living, work-related activities, leisure and relaxation, and social activities. • Sessions included education, group activities and homework assignments completed between the sessions. • Sessions focus on the past, present and desired activities; and homework related to performing the desired activity in a real-life context to test the strategies. • Goals and strategies may be renegotiated through self-analysis, reflection and peer support. • The goal is to reach and maintain a desired balance in daily activities. • The BEL group was facilitated by two therapists (one had BEL training) • Fidelity was self-rated by the facilitators.	• BEL was effective in improving engagement in doing, activity engagement and balance, psychosocial functioning, QOL, and symptoms. • The CAU group also improved in all outcomes, except activity level.	• BEL may be effective for improving functioning and activity engagement. • BEL is time effective. • External validity due to drop-out and lack of blinding may be limited. • The POES provided immediate feedback to participants that can help awareness on their time-use; and is a useful tool in time-use interventions.
Eklund et al. (2023) (66)	Sweden; outpatient and community-based settings (see 47)	To explore implementation of the BEL intervention; and therapists’ views on usefulness within a multi-professional team	Qualitative telephone interviews and content analysis	Occupational therapists (n=13); and managers (n=3) from various multiprofessional teams	Individual and/or joined semi-structured telephone interviews on the process and context of BEL, recruitment, fidelity and target groups.	The BEL (see 47)	• Conditions and opportunities (e.g. expectations of OT interventions and potential benefits of BEL; OTs’ confidence and collaborations; positive and negative influence of the context). • Putting the BEL intervention (e.g. communication, recruitment, retention, adjustments to BEL, and time-use). 1) Experiences of BEL (e.g. specialized OT intervention, goal setting related to everyday life, structure and flexibility of the BEL, participants’ commitments and outcomes)	• BEL is occupation-based and recovery-oriented with homework assignments that helped engagement. • Findings were predominantly from OTs than managers and may be specific to the study setting. • Further instructions for goal setting would be beneficial as participants did not always complete the homework tasks.
Hitch et al. (2022) (64)	Australia; CCUs, PARC and community mental health teams	To describe outcomes of a group program (P2P) to enable activity participation	Descriptive pilot longitudinal design	Adults with menta illness and psychosocial disability (n=17); 11 completed treatment and 8 completed follow-up	• Socio-demographic data • CANAS-P to assess needs over the past month in 22 domains of daily activities. • Time-use diary to measure time-use (including where and with who) for a yesterday with 1-hour intervals. • RAS-DS to measure recovery; • The BASIS-24 to measure psychosocial health and functioning; • LCQ to measure social participation; Completed at baseline, after the program and at 3-month follow-up.	P2P Program: • Developed in consultation with OTs. • To enable engagement in meaningful activities, activity balance and community participation. • Combines the AOI and the Works program (67) • Includes 10 weeks (first 4 weeks AOI, and 6 weeks based on the Works) • 2-hour sessions; consumers were encouraged to attend every session, but was not mandatory. • P2P includes a workbook that scaffolds activities from simple to more complex. • Delivered in a group by OTs and peer-support workers who completed P2P training and regular briefings and reflections	• Decrease in unmet and overall needs. • Increased participation in activities of daily living, productivity and leisure, but time spent in community activities remained unchanged. • Overall increase in recovery scores at follow-up. • No significant changes in psychosocial health. • Small increase in work participation and self-rating of wellbeing but not statistically significant.	• Structured follow-up with the facilitators and case managers helped the positive outcomes. • The P2P is suggested to be delivered by OTs. • External validity may be limited due to small sample size and drop-out; and recruitment from one catchment area in Melbourne.
Hultqvist et al. (2019) (68)	Sweden; outpatient and community-based settings	To explore influence of care context, socio-demographic, clinical and self-related factors in predicting improvements in occupational engagement and QOL following the BEL intervention	Longitudinal study (part of a larger RCT; 47)	See 47	The measurements are the same as Eklund et al., (47) plus: The Pearlin Mastery Scale to estimate self-esteem and self-mastery v)	The BEL (see 47)	• BEL was effective in both community and clinical settings; and with diverse range of participants regardless of socio-demographic, clinical and self-related characteristics. • At 6-month, none of the measures were found as predictors. • At 18-month follow up, psychosocial functioning, friendship, depression and anxiety symptoms, female gender, age, and having children were the strongest predictors of change in occupational engagement and balance. • Self-mastery was negatively associated with changes in occupational balance in leisure domain.	• Encouraging friendship and belonging in the BEL program can enable improvement occupational engagement. • External validity is limited due to skewed sample, non-participants and high drop-out
Lund et al. (2019) (69)	Sweden; outpatient and community-based settings	To explore processes of making changes from participants’ experiences	Qualitative grounded theory design (part of a larger RCT; 47)	Participant in BEL (n=14)	• 29 semi-structured interviews completed after the BEL intervention about person’s background and experiences with BEL, and processes of making changes	The BEL (see 47)	Main category: ‘Breaking a cycle of perceived failure’ that included change processes towards a more balanced lifestyle: 1) Going gently; change is an ongoing process (e.g. identifying strategies, breaking down goals, being kind to oneself). 2) Supports for progress and permission to fail (e.g. supports and strategies for change, daring to try). 3) Prioritizing and setting boundaries (e.g. structuring and setting limits, taking control). 4) Adjusting for a sustainable balance (e.g. pacing oneself, coming to terms with oneself). 5) Caring for a valued self (e.g. utilizing resources, befriending self).	• Making change is an ongoing process with small steps and adjustment. • BEL provided a supportive structure to help participants identify their values and take action towards a balanced lifestyle. • Participants may have had a positive bias in favor of the BEL, and were limited to Swedish context. • Application and exploring usefulness of the BEL in different population would be beneficial.
Lund et al. (2019) (70)	Sweden; outpatient and community-based settings	To explore participants’ experience of meaning of participating in the BEL group	Qualitative grounded theory design (part of a larger RCT; 47)	Eight of the 14 settings were selected from the larger study to recruit participants (n=19) from 10 BEL groups using theoretical sampling	• 26 interviews were completed within 1 week to 6 months after completion of the BEL. • Interviews used open-ended questions on meanings, experiences and views about the group. • Interviewers were blind to the BEL content.	The BEL (see 47)	Meaning making through group participation, connection and support: 1) Joining with others: from alone to connected; from fears and isolation to socialization; not feeling alone with daily-life struggles. 2) Sense of belonging, feeling supported and understood: sharing in a safe context; good and bad days; group acceptance and understanding; expanding social network; bonding and healing through humor 3) Re-valuing self in a more positive way: facing old views and prejudices; reassessing perspectives (e.g. internalized stigma); feeling valued; respecting self and competencies; purpose and self-worth through helping others.	• Joining with others, belonging and universality are important elements of group process that can be enhanced through mutual support and supportive social environment. • Recruitment was through the group leaders which may have been coercive. • Findings may not be applicable to diverse communities as participants from a non-Swedish background were underrepresented (due to language barriers).
Lund et al. (2020) (71)	Sweden; outpatient and community-based settings	To gain an understanding of group leaders’ and participants’ perspectives of the BEL intervention (what helped, hinders or can be improved)	Qualitative grounded theory design (part of a larger RCT; 47)	Group leaders (n=12; including 10 OTs and 2 co-leaders) who have completed at least one BEL group. Participants (n=19) from 10 BEL groups.	• Focus groups for group leaders. • 29 interviews were completed within 1 week to 6 months after the BEL intervention, plus mid-course interviews for 4 participants, and follow-up interviews to test the theory	The BEL (see 47)	Content and format: • Group leaders and participants appreciated the BEL structure and content relevant to daily life, but desired flexibility to meet individuals’ varying needs (e.g. some tasks needed to be adjusted to participants). • Making connections – BEL as a bridge (e.g. closed group structure to share personal experiences; connection to a future life). vi) Facilitating and hindering factors: • Group leaders: 1) Facilitating factors: relevant assessment and evidence-based manual, positive experiences of the participants. 2) Hindering factors: limited space or facilities, and group dynamics. • Participants: 1) Facilitating factors: group dynamics, goal setting and supporting change, feeling connected, competent facilitators, similar experiences with peers, and safe environment. 2) Hindering factors: differences in functioning, previous negative experiences, not having enough time in day-to-day life to implement personal goals or strategies learnt in BEL.	• To consider more flexibility to tailor the group to individuals’ unique and varying in the revised BEL manual. • Need for increasing the intervention time (e.g. to 15 weeks). • Consideration of the environment and resource planning (e.g. space or local trips) would be important. • Group leaders’ communication skills are important in implementing the interventions. • Results may not be applicable to diverse population as participants were limited to Swedish contexts (Due to language barriers).
Rees et al. (2021) (72)	Australia; CCUs	To understand participants’ and facilitators’ viewpoint about the use of the AOI in CCU settings	Qualitative naturalistic design	People with schizophrenia and schizoaffective disorders participate in the AOI (n=10), and group facilitators (n=5)	In-depth semi-structured dividual interviews about participants’ activity patterns, recovery, goals and experiences of the AOI such as impacts on their routines and activity engagement.	The AOI: • Delivered in group format (3 groups overall) by an OT who completed AOI training. • Included 5-8 group sessions. • A facilitator guide was developed from the AOI manual relevant to the local setting.	Participants’ experiences – Making change: • It’s hard to get myself going and things get in the way; • Getting myself going; • Recognizing the value of meaningful activities; • Doing things bring a sense of hope and recovery; Facilitators’ experiences – Facilitating change: • Recognizing inertia as a challenge; • Challenges of getting people going; • Getting people going; • How AOI works to impact inertia.	• Engagement in activities identified by participants can promote recovery. • AOI helped participants to identify barriers to participation and aim for an active living through reflection on their time-use and wellbeing. • AOI offers a flexible and individualized structure. • Findings from this small-scale study may not be applicable in other settings.
Eklund et al. (2023) (66)	Sweden; Community mental health settings	To compare the BEL and TAU group in motivation for engaging in day centers, occupational engagement and personal recovery	RCT (Part of a larger study (47)	Adults with psychiatric disability attending day centers randomly allocated to BEL (n=4) and TAU (n=7) groups	• Socio-demographic questionnaire • A 4-item scale developed for this study to measure motivation (e.g. attending day center, clear goals, time-use and employment preferences). • POES to assess occupational engagement. • QPR to measure personal recovery. • Satisfaction with the day center and quality of support.	The BEL in 14 session over 16-19 weeks (see 47)	• No significant differences between groups in motivation scores. • BEL group improved in occupational engagement and personal recovery.	The BEL offers a novel approach to mental health services that can encourage engagement in meaningful life activities

ACT, Assertive Community Outreach; AOI, Action Over Inertia; SMI, Severe Mental illness; BEL, Balancing Everyday Life; CAU, Care as usual; Oval-pd, Occupational Value with predefined items; MANSA, Manchester Short Assessment of Quality of Life; QOL, quality of life; GAF, Global assessment of functioning; CCU, Continuing care units; PARC, Prevention and recovery care services; P2P, Pathway to Participation; CANSAS, Camberwell Assessment of Need Short Appraisal; RAS-DS, Recovery Assessment Scale—Domains and Stages (RAS-DS); BASIS-24, Behavior and Symptom Identification Scale; LCQ, Living in the Community Questionnaire; QPR, Questionnaire about the Process of Recovery.

#### 3.4.1 Development, content and structure of the time use interventions

The three identified time use interventions are grounded in knowledge that what people do in everyday life matters for health and wellbeing; informed by recovery-oriented practice principles; and draw on educational and cognitive behavioral strategies (14, 62–64). Broadly, they aim to enable engagement in meaningful activities through a combination of educational, activity-based, group or individual sessions, and practice assignments related to engagement in real-world activities. Each uses a manual or participant workbook. They also each emphasize reflection on time use patterns as a means to develop understanding of health and well-being related aspects of activity engagement, and to bring about desired changes in activity patterns.

Action Over Inertia (AOI) (62, 63) was developed by Canadian occupational therapists as a person-centered, strengths-focused and flexible workbook-based approach for supporting individuals to construct activity patterns that enable fulfilling lives. The workbook provides resources, including time diaries, to support individuals to reflection on personal activity patterns and development of strategies for enabling rapid activity changes, supported planning for change, and ongoing supports for sustainable changes (65). More recently, AOI in group formats with 4-10 sessions have been described (63, 72). Integrating the AOI approach with a manualized vocational program, The Works (67), the Pathway to Participation (P2P) group program is designed to scaffold support for activity participation and build momentum for group members to engage in more complex and demanding occupations over time (64).

Balancing Everyday Life (BEL) (47) is a manualized group-based lifestyle intervention designed for people using community-based mental health services. Developed by Swedish occupational therapists, it aims to facilitate engagement in desired activities identified by participants and support for personal recovery (47). Of longer duration than AOI and P2P group programs, the BEL intervention consists of 14 group sessions over 16 weeks and addresses themes relevant to everyday life and personal recovery, including activity balance, meaning and motivation, healthy living, work-related activities, leisure and relaxation, and social activities (14, 66). Each BEL session includes an educational component, a group activity and a home assignment to try out preferred activities and strategies.

All ten studies reported the time use interventions were facilitated by at least one occupational therapist; only P2P groups were co-facilitated with peer or lived experience workers. All reported facilitator training and regular briefings (47, 64, 65).

#### 3.4.2 Experiences and effectiveness of the time use interventions

All ten studies of time use interventions reported positive outcomes. The BEL has been evaluated in rigorous RCT studies that demonstrate it is effective in improving activity engagement, as represented in increased activity levels and more optimal activity balance, and improving personal recovery, clinical symptoms, psychosocial functioning and general quality of life (47, 68). Eklund et al. (47) also reported significant improvements in the control group in activity engagement, satisfaction with activities, symptoms, functioning and general quality of life. The authors suggest this may be attributed to the use of the POES as an outcome measure since it encourages reflection on current activity patterns and their impact on wellbeing, which may itself be a strong agent for change and promote activity engagement independent of the intervention.

A pilot RCT of AOI delivered individually over 12 weeks also showed small but not statistically significant activity changes: decreased time spent sleeping and increased general activity compared to a control group (65). The 10-week P2P group program was evaluated using a non-randomized, longitudinal study design. It too reported improvements in self-rated recovery scores and reduction of unmet needs through activity engagement, but no changes were detected in time use or psychosocial health measured using the Behavior and Symptom Identification Scale (BASIS-32) (64).

Five qualitative studies explored participants’ and facilitators’ experiences of the time use interventions; four of which related to the BEL (**Table 4**). Overall, participants reported the BEL program structure and content tailored to their skills and interests were helpful, along with opportunities to connect with peers and share life experiences in a supportive environment (69–71) This suggests connectedness and belonging were central to participants’ experiences of the BEL intervention (70). The group components also encouraged reflection, envisioning and connecting to a more positive future, and sustaining change (69–71). However, participants suggested more flexibility to tailor the program to their needs, and more time and support for goal setting and implementing strategies in daily life would be helpful (71). The BEL group facilitators also perceived the program structure of the program as beneficial, along with its alignment to occupational therapy through activity engagement and promotion of collaboration within multidisciplinary teams (71). From their experience, the main barriers in implementing BEL were variation in individual needs and challenges, limited space or information technology issues (71).

Regarding AOI groups, Rees et al. (72) also reported participants’ experiences of time use were changed by reconnecting with the value of meaningful activities and addressing barriers to sustaining activity engagement. The facilitators reported the concept of ‘inertia’, as conceptualized in the AOI, helped to identify restricted time use patterns and participation barriers as potential drivers for change. Rees et al. (72) also highlighted the value of peer learning and support when addressing time use, and recommended peer worker involvement in AOI group facilitation.

## 4 Discussion

This scoping review aimed to synthesize evidence addressing the review question: *‘How does a time use perspective inform assessment and interventions in community mental health practice?’*

The findings identified applications of a time use perspective to develop methods of assessment, to measure the effectiveness of interventions, and as a basis for structured interventions in community mental health practice. Here considerations for their further use are discussed.

### 4.1 Time use assessment and outcome measures

Measuring time use is complex given the diversity of human activity and how multiple simultaneous activities or activities with intermittent interruptions occur across time (73). This review identified three approaches to time use assessment and evaluation currently being used: 1) measures collecting data at fixed intervals using pre-determined activity categories (e.g. TUS); 2) measures collecting data at fixed intervals with activities defined by people with severe mental illness themselves (e.g. POES); and 3) multiple daily spot sampling methods for capturing activity data in real time (e.g. ESM) (73, 74), as explored using smart phone technology (e.g. 41, 42).

Categorizing time use according to predetermined categories of activities has a long history in public health and social sciences for understanding human behavior in a temporal context; determining activity patterns and balance; identifying time use trends in structured or economically valued activity; and as an indicator of wellbeing or quality of life (4, 74). This approach is reflected in Time Use Survey (TUS) instruments, originally developed for population studies, and used to evaluate outcomes in six intervention studies identified in this review. These studies evaluated differing psychosocial and occupational interventions and indicate that the TUS is a relevant and sensitive measure, suitable for evaluating interventions designed to provide individualized support for activity engagement broadly or for participation in specific domains such as employment or socialization. The use of TUS offers advantages of well-understood language for reporting activity categories to enable communication of study results and comparability between studies (74). It also offers opportunities to consider the time use of people with severe mental illness in relation to that of the general population (74) and also specific groups within the general population (e.g. those not in the workforce, retirees, carers), as part of a more nuanced approach to analysis of activity patterns and disruptions (62, 63).

There are drawbacks to the application of TUS for time use assessment and evaluation in mental health practice. For instance, pre-determined activity categories have limited use to understand aspects of activity experiences, like their variety, meaningfulness, satisfaction and social aspects (e.g. where and with whom activities were undertaken). These aspects contribute to a holistic understanding of person’s time use, including activity experiences within temporal and social contexts, that is necessary to illuminate the interacting biological, psychological and social forces in which many activity disruptions experienced by people with severe mental illness are situated (63). An alternative approach to time use assessment, such as the POES (36), focuses on activities as defined by people with severe mental illness themselves and is more able to evaluate a range of health and well-being aspects of activity engagement that are sensitive to the influence of complex factors like capacities, experiences, and the course of recovery (36, 37, 48, 51). In so doing, POES highlights the extent to which a person’s activities are aligned with their values, capabilities and resources (14). As indicated by the studies in this review, this means POES is a useful tool for assessment and evaluating outcomes of importance to both personal recovery and community inclusion.

The time use assessments identified in this review also appear to be valuable and inexpensive tools for reflection on one’s time use, health and well-being through activity, developing awareness of personal needs and issues, describing the frequency and experiences of related behaviors, identifying the impacts of symptoms, treatments and resources in daily life, and informing goal planning (36, 37, 39). However, further evidence of the potential of time use based reflective tools to support self-determination, and to enable individuals to self-monitor their time use in relation to health and wellbeing, as part of recovery oriented and self-management approaches needs development (75). With activity-related on-line information and resources, tracking devices and other applications continuing to advance, the ESM approaches are particularly well suited for integrating time use information into person-centered recovery-oriented care and self-management tools, such as the Wellness Recovery Action Plan (WRAP) (76). Yet, the needs of people with severe mental illness for sustainable and equitable access require further consideration to overcome the ‘digital divide’ posed by socioeconomic circumstances (77).

Some limitations of time diary-based assessments, such as POES (36) and the simplified time budget (39), should be noted. While they typically include a retrospective interview that offers opportunities for practitioners to explore perceptions of activity engagement and time use, difficulties related to recall may be more pronounced for individuals with severe mental illnesses due to cognitive issues, lack of daily prompts or routines, or issues related to reporting particular activities. The most recent version of Action over Inertia (63) prompts practitioners to consider unreported daily activities, such as work for informal payments, using or selling drugs, begging, and other forms of activities that may have personal value but can also adversely impact health and wellbeing. Spot sampling methods with multiple daily reminders (e.g. ESM) can overcome recall challenges and offers more flexibility in measuring activity duration (73, 74), but issues with low adherence and accuracy of responses over an extended period (over 5-6 days), technical difficulties with devices, and potential exacerbation of positive symptoms are also reported (20, 41, 42). These are important issues for which engaging lived experience and professional perspectives in co-designing solutions will benefit future developments.

### 4.2 Interventions

While daily time use is a highly personal experience, influenced by various factors embedded in larger environmental contexts (e.g. culture, income, neighborhood structures, family expectations), the interventions identified in this review explicate the knowledge base and standardized processes underlying practice for addressing health and well-being aspects of time use collaboratively with people with severe mental illness. They contribute to emerging evidence on interventions supporting personal recovery (78–80). The effectiveness and external validity of these intervention may be limited. For example, most studies reported small number of participants, issues with retention and work conducted in specific contexts (e.g. Swedish mental health services). The authors of the Randomized Control Trial studies acknowledged that lack of blinding may have also impacted the findings (14, 65).

Ongoing advancements in the field are leading to the inclusion of a broader range of factors that influence implementation of interventions in practice. The BEL, AOI and P2P interventions utilize either group or individual session formats that can be modified or adapted to individuals’ needs and preferences. They also all include homework or activity experimentation based on personal goals, aligned with current knowledge regarding the transference of choices made in therapeutic settings to the real-world, including the importance of ongoing support, evaluation and adaptation (81). While peer learning and support were also identified as beneficial aspects of the group programs (70, 72), only P2P involved lived experience co-facilitators. Given emerging evidence that peer involvement in developing and delivering social interventions contributes to positive outcomes (82), this is an important avenue for further development of time use focused interventions.

Thinking ahead, the field may benefit from advancing and implementing conceptual frameworks to systematically link time use activity patterns with personal recovery, health and well-being for people with severe mental illness. This would ensure shared definitions, demonstrate the links between activity patterns and health and well-being, support the evidence base, and serve as a foundation for comparing measurement tools and intervention approaches. For example, the Do-Live-Well Framework (DLW) (83–85), a conceptual framework from Canada, identifies eight activity experiences and five activity patterns previously shown to impact health and well-being. Integrating such a framework in the mental health field depends on further study of its relevance to people with severe mental illness living in the community, such as aligning to their goals and preferences.

Ensuring that the complex factors influencing time use are systematically considered in intervention approaches depends on integrating theory and conceptual models into their design, as well as lived experience and professional perspectives. The International Classification of Functioning, Disability and Health (ICF, 86), recovery frameworks (80, 87, 88), and understandings of cognition and motivation provided the foundation of time use scholarship and practice in the mental health field. With the growing understanding of the social forces that marginalize or constrain activity participation for people with severe mental illness, these must be addressed in any intervention approach that purports to focus on supporting community inclusion and quality of life (89). Hence, the updated version of AOI (63) includes identification of transportation, stigma, discrimination and personal safety related barriers. However, to consider environmental, social and other contextual factors more seriously, other theories and perspectives are needed. For example, perspectives such as Occupational Justice (90) and the Capabilities Framework (91, 92) ground an understanding of what people can do and be in the structural and contextual conditions that support or restrict possibilities for doing, and thereby could locate time use scholarship and practice in the realm of social justice.

### 4.3 Limitations

This scoping review focused on applications of a time use perspective in community mental health practice involving people with severe mental illness. Hence, the reviewed time use measures and interventions may not be applicable to other populations or in inpatient settings. Since the time use studies of people with mood or anxiety disorders and/or high prevalence mental illness were excluded from this scoping review, these warrant further exploration (93). Most reviewed studies were conducted in North America, Europe or Australia, so that the findings may not be generalizable to other cultural or geographical contexts. The heterogeneity of study methodologies, limited longitudinal data and flexibility of intervention formats and delivery were barriers to consolidating this evidence and commenting on the sustained impact of interventions. Inclusion of stakeholder consultation is recommended to enhance methodological rigor (32). While this scoping review was informed by a team with perspectives as researchers, educators and mental health clinicians, consultation with other stakeholders, such as people with severe mental illness or peer workers in mental health services may have enhanced synthesis of this research.

## 5 Conclusions

This scoping review described practice applications of a time use perspective in community mental health practice. It identified time use assessments that evaluate outcomes of importance to personal recovery and community inclusion; and time use interventions designed to address activity patterns associated with poor health and well-being. These approaches emphasize reflection, collaborative goal planning and supported activity engagement, and contribute to emerging evidence on interventions that support self-management and personal recovery. While most time use assessments and interventions originate in occupational therapy, other mental health disciplines have begun to measure time use as a means to evaluate psychosocial intervention outcomes. This is an important step in raising the profile of time use perspectives across the mental health field and integrating time use approaches into evidence informed practices within community mental health services.

## Data Availability

The original contributions presented in the study are included in the article/**Supplementary Material**. Further inquiries can be directed to the corresponding author.
