# Supplementary material for: Contributions of a time use perspective in community mental health practice: a scoping review
Source: Front Psychiatry. 2024 Oct 11;15:1461705. doi: 10.3389/fpsyt.2024.1461705 (PMC11502927; doi:10.3389/fpsyt.2024.1461705)
Supplement: Supplementary file 1 [file Table1.docx]

**Table S1 Supplementary; search strategy**

| **Category** | **Search terms** |
| --- | --- |
| Setting and population | "Mental disorder*" OR "Psychotic disorder*" OR "Schizophreni*" OR "Mental illness*" OR "Psychosocial disabilit*" OR "Psychiatric disorder*" OR "Psychiatric patient*" OR "Mentally ill" |
| Perspective | All adults (included as a ‘limit’ to filter the search) |
| Phenomenon of Interest | "Time-use" OR "time-use" OR "Use of time" OR "Occupational balance" OR "Occupational engagement*" |
| Comparison | Not applicable |
| Evaluation | Peer reviewed journal articles including quantitative, qualitative and mixed methods studies reporting primary research |
